# Illuminating a new path to multicellularity

**DOI:** 10.7554/eLife.83296

**Published:** 2022-10-11

**Authors:** Sayantan Datta, William C Ratcliff

**Affiliations:** 1 Interdisciplinary Graduate Program in Quantitative Biosciences, Georgia Institute of Technology Atlanta United States; 2 School of Biological Sciences, Georgia Institute of Technology Atlanta United States

**Keywords:** origin of multicellularity, two-dimensional nematic liquid-crystal, ecological scaffolding, limestone cave, competition-dispersal trade-off, evolution, Other

## Abstract

A new species of multicellular bacteria broadens our understanding of prokaryotic multicellularity and provides insight into how multicellular organisms arise.

**Related research article** Mizuno K, Maree M, Nagamura T, Koga A, Hirayama S, Furukawa S, Tanaka K, Morikawa K. 2022. Novel multicellular prokaryote discovered next to an underground stream. *eLife* **11**:e71920. doi: 10.7554/eLife.71920.

Billions of years before eukaryotes took their first multicellular steps, prokaryotes such as bacteria and archaea began exploring their own multicellular lifestyles. These included chain-forming cyanobacteria that formed softball-sized spheres, packs of bacteria hunting mobile prey together, and filamentous bacteria dividing labor to saturate their growth substrate with antibiotics to protect their aerial spores from threats as they develop (Dodds et al., 1995; Fortezza et al., 2022; Zhang et al., 2020).

Fossils of large, strangely shaped colonial organisms and tunnel systems point to a renaissance of prokaryotic multicellularity around two billion years ago, during a short period of elevated oxygen (El Albani et al., 2010; El Albani et al., 2019). Over time, countless other prokaryotes have evolved transient multicellular phases in their life histories, allowing them to gain some of the benefits of multicellularity without fully committing to this strategy (Shapiro, 1998). Now, in eLife, Kouhei Mizuno (National Institute of Technology), Kazuya Morikawa (University of Tsukuba) and colleagues at various institutes in Japan report on a previously unknown form of bacterial multicellularity (Mizuno et al., 2022).

Deep in a limestone cave system in northern Kyushu Island, Japan, growing alongside a river that periodically fills the cave chamber, they discovered a new species of bacteria, *Jeongeupia sacculi* sp. nov. HS-3. This bacterium forms well-ordered sheets of cells, which ultimately develop into a bulbous structure that actively releases germ-like, single celled propagules when submerged. This type of multicellularity has little precedent, though it somewhat resembles puffball fungi, which also form propagules within an enclosed multicellular structure, ejecting them into the environment when mechanically disturbed.

When grown in the laboratory, most bacteria multiply in a disordered manner and form opaque colonies. HS-3, in contrast, built transparent ones with an iridescent hue. Further investigation revealed that HS-3 multiplied in a highly regulated manner, first creating short, pill-shaped cells (termed coccobacilli), which then elongated to form a single layer of tightly packed filamentous cells. Mizuno et al. showed that the unusual appearance of HS-3 was due to the regularity of their cellular packing: HS-3 cells lined up in parallel, forming a nematic liquid crystal. For the next two days, the colonies continued to increase in size and to add layers of packed, almost coiled, filamentous cells. They then ceased growing, with no apparent change for another next two to three days.

But HS-3 was not idle – it was simply shifting into the reproductive phase of its life cycle. In the center of the colony, between stacks of tightly packed filamentous cells, HS-3 began to form large numbers of coccobacilli. When immersed in water, the coccobacilli were rapidly ejected from a pore in the colony’s center. Given the location of their discovery, Mizuno et al. infer that HS-3 colonies are regularly immersed in water when the cave floods; coccobacilli propagules would then be dispersed by the river, some presumably would find new patches of cave wall to inhabit, and the multicellular life cycle would start again (Figure 1).

**Figure 1.**
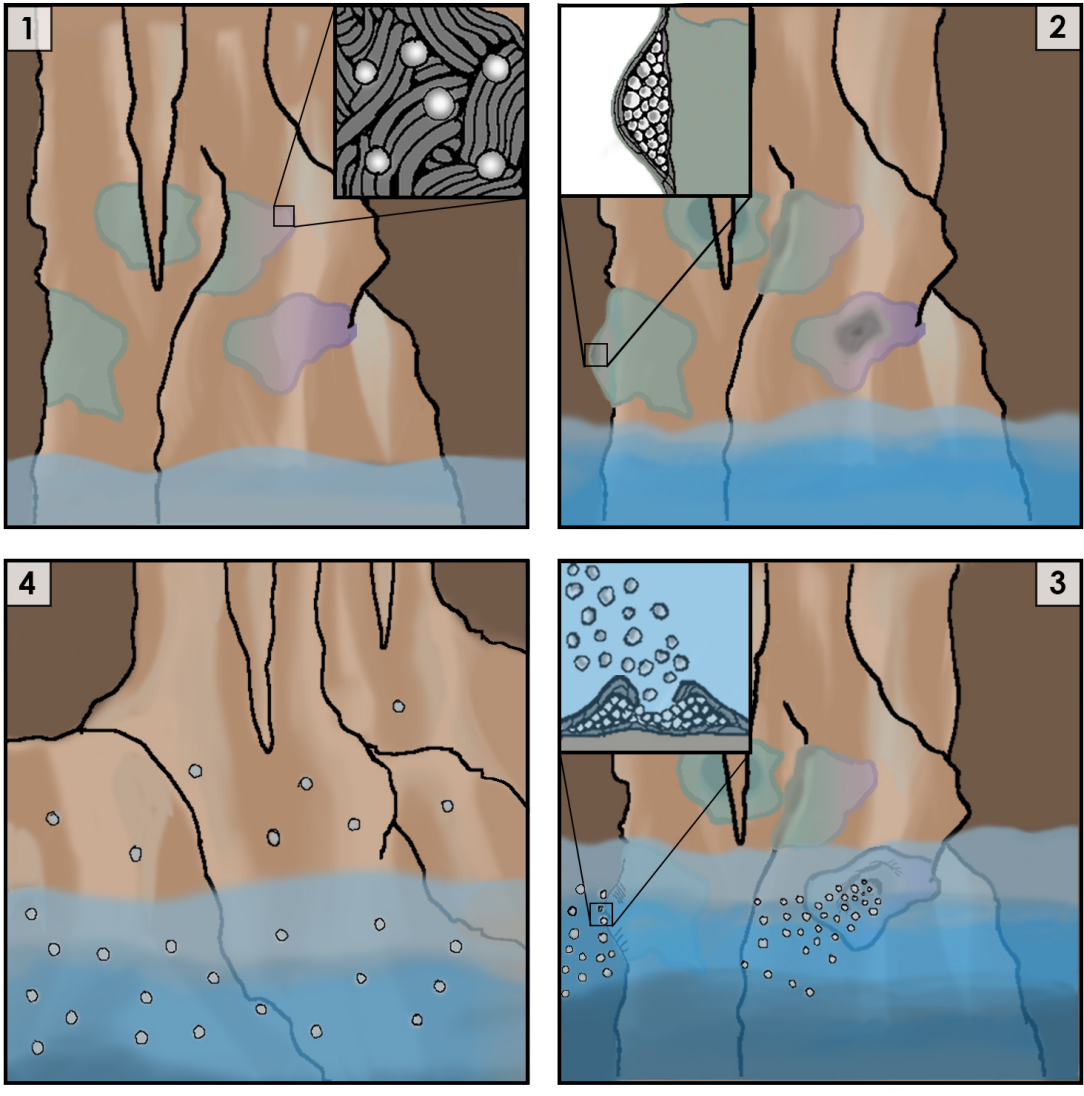
Cartoon representation of the life cycle of Jeongeupia sacculi sp. nov. HS-3's in an underground cave system. [1] The bacterium grows on cave walls in flat, liquid crystal colonies (Inset: colony morphology at expanding edge). [2] Production of coccobacilli causes the colonies to swell (Inset: cross-section of a colony showing coccobacilli stored internally). [3] Rising river levels submerge the colony, causing coccobacilli to be released (Inset: coccobacilli streaming from the colony center). [4] Coccobacilli are dispersed by water currents and deposited on cave walls, starting the life cycle over again.

The work of Mizuno et al. helps us to understand how multicellularity can evolve. The first step in this transition is for groups of cells to become distinct evolutionary units (Ratcliff et al., 2012). Once groups are able to reproduce and possess heritable variation in traits that affect their fitness, they can start to acquire group-level adaptations that allow them to become more integrated and organismal (Rose and Hammerschmidt, 2021).

The ecological scaffolding hypothesis argues that multicellular life cycles, which allow groups to become evolutionary units, could have first been created by the environment rather than being encoded developmentally within the life cycle of a species (Black et al., 2020). So far, however, we have been unable to determine if ecological scaffolding has played a role in the evolution of multicellularity, as we know little about the environmental conditions in which most multicellular lineages first evolved.

HS-3, in contrast, is a strong candidate for ecological scaffolding. Before multicellularity evolved in this species, single cells deposited on a cave wall by receding flood waters could have divided into clonal colonies. These would have been redispersed by the next flood, generating a simple multicellular life cycle. It is easy to imagine how mutations affecting multicellular development could have been selected for under these conditions, gradually paving the way for genetically-regulated developmental control.

Whether prokaryotes such as HS-3 truly are multicellular has long been a source of controversy (Shapiro, 1998), in part because ‘multicellularity’ is a poorly defined term. Using the presence of specific traits to define this evolutionary transition often fails to be sufficiently general; instead, it may be more accurate to use evolutionary dynamics as an indicator. Specifically, when groups of cells gain adaptations due to group-level selection, they can be considered a multicellular individual (Rose and Hammerschmidt, 2021). Key traits of HS-3, including coordinated colony growth, differentiation, and dispersal upon submersion, all appear to be multicellular adaptations to a reliably fluctuating environment – though Mizuno et al. did not formally test adaptive hypotheses.

People often ask why prokaryotes, which unambiguously evolved multicellularity billions of years before eukaryotes, nonetheless remained ‘simple’. We should not be too quick to accept this premise. As this remarkable study demonstrates, prokaryotic multicellularity may often be context-dependent, and not immediately obvious to an observer. Prokaryotes represent a vast and relatively untapped reservoir of multicellular innovation, whose study can shed light on the diverse routes to increasingly complex life. Who knows what strange, remarkable multicellular creatures may be lurking in the deep dark recesses of the world, awaiting discovery?
